# MiR-146b-3p protects against AR42J cell injury in cerulein-induced acute pancreatitis model through targeting Anxa2

**DOI:** 10.1515/biol-2021-0028

**Published:** 2021-03-16

**Authors:** Kunpeng Zhang, Xiaoyu Zhang

**Affiliations:** Department of Hepatobiliary Surgery, Xingtai People’s Hospital, Xingtai, Hebei, 054001, China; Department of Neurology, Xingtai People’s Hospital, 16 Hongxing Street, Qiaodong District, Xingtai, Hebei, 054001, China

**Keywords:** acute pancreatitis, miR-146b-3p, Anxa2, AR42J, cerulein-induced acute pancreatitis

## Abstract

**Background:**

Acute pancreatitis (AP) is a common inflammatory disorder. MicroRNAs play crucial roles in the pathogenesis of AP. In this article, we explored the detailed role and molecular mechanisms of miR-146b-3p in AP progression.

**Methods:**

The rat AR42J cells were treated with cerulein to establish the AP model *in vitro*. The miR-146b-3p and Annexin A2 (Anxa2) mRNA levels were assessed by quantitative reverse transcriptase-polymerase chain reaction (qRT-PCR). Cell viability and apoptosis were tested using the Cell Counting Kit-8 (CCK-8) and flow cytometry assays, respectively. Caspase-3 activity and the production of interleukin-1β (IL-1β), IL-6, and tumor necrosis factor-α (TNF-α) were measured by enzyme-linked immunosorbent assay and qRT-PCR. Targeted interaction between miR-146b-3p and Anxa2 was verified by the dual-luciferase reporter and RNA immunoprecipitation assays. Western blot analysis was performed to detect the expression of Anxa2 protein.

**Results:**

Our data revealed that miR-146b-3p was significantly downregulated in AP samples. The enforced expression of miR-146b-3p alleviated cerulein-induced injury in AR42J cells, as evidenced by the promotion in cell viability and the repression in cell apoptosis, as well as the reduction in IL-1β, IL-6, and TNF-α production. Anxa2 was directly targeted and inhibited by miR-146b-3p. Moreover, the alleviative effect of miR-146b-3p overexpression on cerulein-induced AR42J cell injury was mediated by Anxa2.

**Conclusions:**

The current work had led to the identification of miR-146b-3p overexpression that protected against cerulein-induced injury in AR42J cells at least in part by targeting Anxa2, revealing a promising target for AP diagnosis and treatment.

## 1 Introduction

Acute pancreatitis (AP) is the most common gastrointestinal disease worldwide [1,2]. Severe AP, accounting for 15–20% of AP cases, leads to a strong systemic inflammatory response and multiple organ failure [3]. Despite advances in diagnostic and therapeutic techniques, AP is still associated with significant mortality [4]. A more precise understanding of the molecular basis of AP pathogenesis is crucial for developing better therapeutic interventions.

MicroRNAs (miRNAs) are small non-coding RNAs, approximately 19–23 nucleotides long, which play important roles in almost all biological pathways [5]. MiRNAs silence gene expression by pairing to the 3′-untranslated region (3′-UTR) of target mRNAs, resulting in translational suppression and target mRNA degradation [6]. Emerging evidence has shown the crucial involvement of miRNAs in various diseases, including AP [7,8]. For instance, Miao et al. uncovered that miR-148a weakened cerulein-induced autophagy in the AP cell model by targeting interleukin-6 (IL-6)/signal transducer and activator of transcription 3 (STAT3) pathway [9]. Wang et al. reported that the reduced level of miR-155 diminished AP’s progression by regulating the Th17/Treg ratio via targeting suppressor of cytokine signaling 3 [10].

MiR-146b-3p has been identified as vital regulators in many cancers, such as cervical cancer, thyroid cancer, and esophageal cancer [11,12,13]. The loss of miR-146b-3p was found to be associated with the risk of age-related vascular diseases [14]. Moreover, it has been reported that the increased level of miR-146b-3p reduced TNF-α production in the amadori-glycated albumin-induced human macrophages by targeting adenosine deaminase [15]. Interestingly, when we used the Gene Expression Omnibus (GEO) database (Accession: GSE61741) to explore the dysregulated miRNAs in AP, we found that miR-146b-3p was significantly downregulated in AP patients compared with the control. Therefore, we undertook to investigate the role and molecular mechanisms of miR-146b-3p in AP pathogenesis.

Annexin A2 (Anxa2), a 36 kDa protein, is a major cell receptor in endothelial cells [16]. Overexpression of Anxa2 in cancer cells has widely been reported to impact human carcinogenesis [17,18]. Previous work also demonstrated the abnormal expression of Anxa2 in cerulein-induced AP cell model [19,20]. Moreover, Zhao et al. showed that Anxa2 enhanced the apoptosis of cerulein-induced AR42J cells, thereby contributing to AP progression [20]. Nevertheless, it is still unclear whether Anxa2 represents a functional target of miR-146-3p in modulating AP pathogenesis.

In this study, we first established the AP cell model *in vitro* using AR42J cells. Subsequently, we explored the precise, critical role of miR-146b-3p in AP progression.

## 2 Materials and methods

### 2.1 Bioinformatics

The dysregulated miRNAs in 37 patients with pancreatitis and 94 normal controls were analyzed using the GEO database (Accession: GSE61741) at https://www.ncbi.nlm.nih.gov/geo/query/acc.cgi?acc=GSE61741. The molecular targets of miR-146b-3p were predicted by the TargetScan v.7.1 software at http://www.targetscan.org/vert_71/?tdsourcetag=s_pcqq_aiomsg.

### 2.2 Clinical samples

In this study, 35 patients diagnosed with AP using high plasma amylase and abnormal pancreatic morphology by ultrasonic diagnosis were recruited from Xingtai People’s Hospital between April 2017 and June 2018. Simultaneously, 10 healthy volunteers were enrolled, who had no inflammatory diseases, tumors, or infections. The clinical characteristics of these participators are provided in Table 1. Peripheral blood was collected from all participants, and serum samples were stored at −80°C.

**Table 1:** Clinical characteristics of AP patients and healthy controls

Characteristic	AP patients	Normal control
Age (years)	53.25 ± 9.33	48.69 ± 7.14
Female (%)	10 (28.6)	4 (40)
Glu (mmol/L)	6.58 (5.67–10.19)	4.92 (4.84–5.21)
TC (mmol/L)	0.62 ± 0.16	0.63 ± 0.07
TG (mmol/L)	1.63 (1.15–3.38)	1.12 (0.92–1.43)
HCT	0.38 (0.27–0.45)	0.44 ± 0.03
Ca^2+^ (mmol/L)	1.98 (1.91–2.08)	—
CRP (mg/L)	135.69 (69.47–201.38)	—
LPS (U/L)	2.83 ± 0.53	—
AMY (U/L)	2.37 ± 0.59	—
Ranson score	2.91 (2.21–3.49)	—

AMY: amylase, CRP: C reactive protein, Glu: glucose, HCT: hematocrit, LPS: lipase, TC: total cholesterol.


**Informed consent:** Informed consent was obtained from all the individuals included in this study.
**Ethical approval:** The research related to human use has been complied with all the relevant national regulations and institutional policies and in accordance with the tenets of the Helsinki Declaration, and has been approved by the Ethics Committee of Xingtai People’s Hospital.

### 2.3 Cell culture

The rat pancreatic acinar AR42J cells were purchased from the American Type Culture Collection (ATCC, Rockville, MD, USA) and maintained in Kaighn’s Modification of Ham’s F-12 Medium (F-12K, Gibco, Tokyo, Japan) containing 10% fetal calf serum (FCS, Gibco) as reported [21]. The cells of ∼60% confluence were exposed to 10 nmol/L of cerulein (Sigma-Aldrich, Taufkirchen, Germany) for 24 h.

### 2.4 Quantitative reverse transcriptase-polymerase chain reaction (qRT-PCR)

Total RNA was obtained from serum samples and AR42J cells using the RNeasy Mini Kit (Qiagen, Hombrechtikon, Switzerland) based on manufacturers’ protocols. Reverse transcription (RT) was performed with 1 µg of total RNA using QuantiTect RT Kit (Qiagen) for mRNAs expression and miScript RT Kit (Qiagen) for miR-146b-3p level. Then, qRT-PCR was carried out using the SYBR Green PCR Master Mix or miScript SYBR Green PCR Kit (Qiagen) on the Rotor-Gene Q instrument (Qiagen). The indicated mRNAs and miR-146b-3p expression were normalized against glyceraldehyde 3-phosphate dehydrogenase (GAPDH) or U6 and calculated using the 2^−ΔΔCt^ method. PCR primer sets for miR-146b-3p and U6 were obtained from Qiagen, and the PCR primers for the indicated human and rat gene mRNAs are provided in Table A1.

### 2.5 Transfection of oligonucleotide and plasmid

For miR-146b-3p overexpression studies, AR42J cells (5.0 × 10^4^) were transiently transfected with the commercial miR-146b-3p mimic (20 nM, GeneChem, Shanghai, China) or a scrambled oligonucleotide sequence (miR-con mimic, 20 nM, GeneChem). For the upregulation of Anxa2 studies, pcDNA-based Anxa2 overexpression plasmid (Anxa2, 10 ng, GeneChem) was transiently introduced into AR42J cells (5.0 × 10^4^), with the nontarget plasmid (pcDNA, 10 ng, GeneChem) as a negative control. Cells were transfected with the indicated oligonucleotide and plasmid using the cationic lipid-based Lipofectamine 3000 reagent (Thermo Fisher Scientific, Vienna, Austria) as per the instructions of manufacturers.

### 2.6 Determination of cell viability and apoptosis

Cell viability and apoptosis were analyzed using the Cell Counting Kit-8 (CCK-8, Dojindo, Kumamoto, Japan) and flow cytometry assays, respectively. Briefly, AR42J cells were transfected with miR-con mimic, miR-146b-3p mimic, miR-146b-3p mimic + pcDNA or miR-146b-3p mimic + Anxa2 overexpression plasmid, and then treated with 10 nmol/L cerulein for 24 h. In viability assays, approximately 10 µL of CCK-8 solution per well was used at 37°C for 2 h, followed by the measurement of absorbance using a Multiskan EX microplate reader (Thermo Fisher Scientific, Runcorn, UK) at 450 nm. In apoptosis assays, the cells were double-stained with 5 µL of Annexin-V-fluorescein isothiocyanate (FITC) (BD Biosciences, San Jose, CA, USA) and 2 µL of propidium iodide (PI, 0.5 mg/mL, Sigma-Aldrich), followed by the analyses of apoptotic data by the FACSCanto II flow cytometry (BD Biosciences) with CellQuest software.

### 2.7 Enzyme-linked immunosorbent assay (ELISA)

Caspase-3 activity was assessed by the Colorimetric Caspase-3 Assay Kit (BioVision, Wehrheim, Germany) as recommended by the manufacturers. The levels of interleukin-1β (IL-1β), IL-6, and tumor necrosis factor-α (TNF-α) were measured by the corresponding Commercial rat ELISA Kit (Thermo Fisher Scientific) based on the protocols of manufacturers.

### 2.8 Dual-luciferase reporter and RNA immunoprecipitation (RIP) assays

The 3′-UTR region of Anxa2 and the mutant in the miR-146b-3p binding sites were individually cloned into the pmirGLO vector (Promega, Charbonnières, France) downstream from the firefly luciferase coding region as reported previously [22]. AR42J cells (5.0 × 10^4^) were cotransfected with 50 ng of each reporter construct and 20 nM of miR-146b-3p mimic or miR-con mimic using Lipofectamine 3000 reagent. Forty-eight hours post-transfection, the cells were analyzed for both firefly and Renilla luciferase activity using the Dual-luciferase Reporter Assay System (Promega).

For RIP assays, the lysates of AR42J cells were prepared using the Complete Lysis-M reagent (Roche Diagnostic, Sussex, UK) and were then incubated with protein A/G beads-conjugated antibody against Argonaute2 (anti-Ago2, #2897, Cell Signaling Technology, Danvers, MA, USA) or negative control isotype IgG (anti-IgG, #3900, Cell Signaling Technology) at 4°C for 4–6 h. The beads were washed twice with ice-cold phosphate-buffered saline, and total RNA was isolated to measure the enrichment levels of Anxa2 and miR-146b-3p by qRT-PCR.

### 2.9 Western blot for Anxa2 protein

Total protein was extracted and western blot analysis was performed as previously reported [21]. Total protein (50 µg) was resolved on sodium dodecyl sulfate-polyacrylamide gels and then transferred onto the nitrocellulose membranes (GE Healthcare, Little Chalfont, UK). The membranes were blocked in 5% non-fat milk and then probed with anti-Anxa2 (ab41803, Abcam, Toronto, ON, Canada; dilution 1:1,000) or anti-β-actin (ab8227, Abcam; dilution 1:3,000) antibody, followed by the incubation with horseradish peroxidase-coupled IgG (ab205718, Abcam; dilution 1:10,000) secondary antibody. Enhanced chemiluminescence Kit (Amersham Biosciences, Freiburg, Germany) was used for the detection of the blot signals with the ImageJ software (National Institutes of Health, Bethesda, MD, USA).

### 2.10 Statistical analysis

All assays were repeated thrice. A two-sided Student’s *t*-test was used for the statistical analysis between two groups. Differences in multiple groups were analyzed by analysis of variance with Tukey’s *post hoc* test. The Spearman test was used to determine the correlation between Anxa2 and miR-146b-3p expression in the serum samples of AP patients. *P* < 0.05 meant a statistically significant difference.

## 3 Results

### 3.1 MiR-146b-3p expression is downregulated and Anxa2 level is upregulated in AP samples

**Figure 1:**
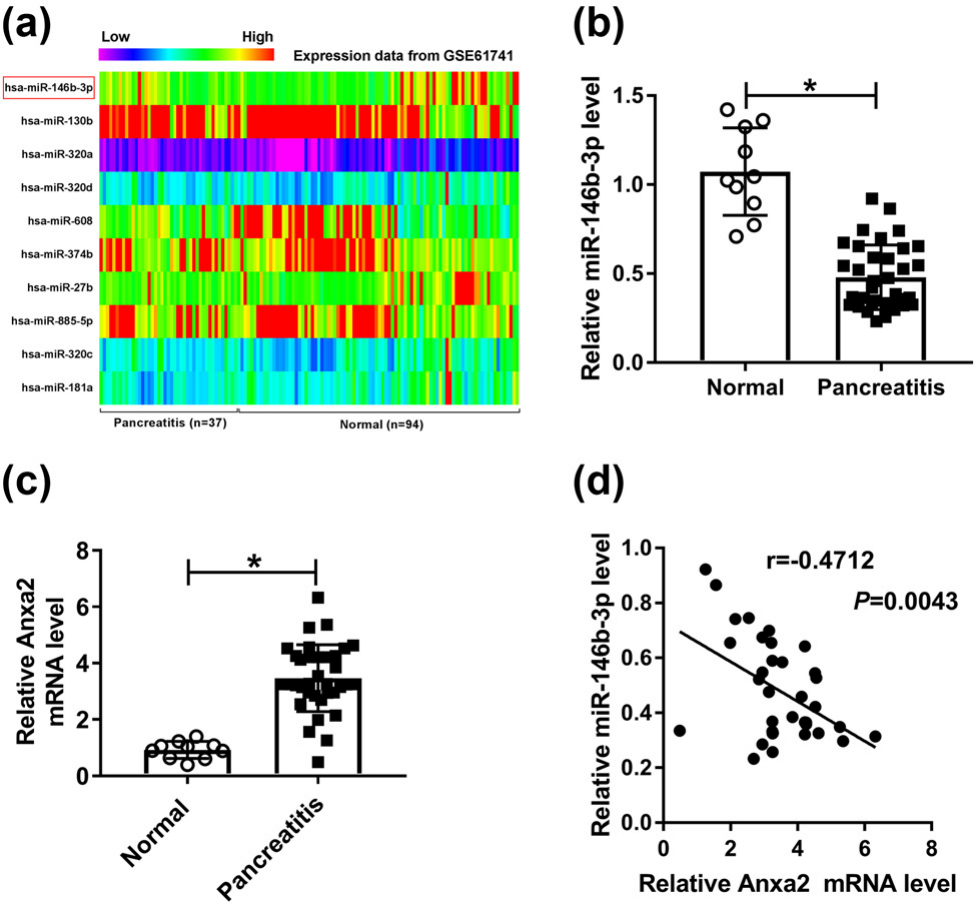
MiR-146b-3p was significantly downregulated in AP samples. (a) Cluster heat map showed the 10 most significantly dysregulated miRNAs in AP samples compared with the normal. (b) MiR-146b-3p level was assessed by qRT-PCR in the serum samples of 35 AP patients and 10 healthy controls. (c) Anxa2 mRNA level was detected by qRT-PCR in the serum samples of 35 AP patients and 10 healthy controls. (d) Correlation between Anxa2 mRNA and miR-146b-3p was evaluated in the serum samples of AP patients using the Spearman test. *P < 0.05.

To preliminarily investigate the involvement of miRNAs in AP, we used GEO database to analyze the aberrant miRNAs from 131 samples, including 37 patients with pancreatitis and 94 normal controls. The heat map data showed the 10 most significantly dysregulated miRNAs, of which miR-146b-3p was prominently downregulated in AP samples (Figure 1a, Table 2). Then, we validated the expression of miR-146b-3p in the serum samples of AP patients. As demonstrated by qRT-PCR, miR-146b-3p level was significantly decreased in the serum samples of AP patients compared with the normal control (Figure 1b). Conversely, Anxa2 mRNA was remarkably overexpressed in the serum samples of AP patients compared with the normal control (Figure 1c). Interestingly, a strong inverse correlation between Anxa2 and miR-146b-3p expression was found in the serum samples of AP patients (Figure 1d).

**Table 2:** The 10 most significantly dysregulated miRNAs in AP samples compared with the normal control using GEO database

ID	Adj. *P*-value	*P*-value	*t*	*B*	log FC
hsa-miR-146b-3p	1.53 × 10^−12^	1.80 × 10^−15^	−9.015158	24.6475	−2.849538
hsa-miR-130b	1.91 × 10^−8^	4.50 × 10^−11^	7.174257	14.866	2.898943
hsa-miR-320a	3.52 × 10^−8^	1.25 × 10^−10^	−6.980039	13.8836	−1.242371
hsa-miR-320d	6.49 × 10^−8^	3.06 × 10^−10^	−6.806564	13.0164	−1.764002
hsa-miR-608	1.04 × 10^−7^	6.32 × 10^−10^	6.665551	12.3192	3.360575
hsa-miR-374b	1.04 × 10^−7^	7.33 × 10^−10^	6.636478	12.1763	2.659228
hsa-miR-27b	1.17 × 10^−6^	1.14 × 10^−8^	−6.08765	9.5403	−2.032959
hsa-miR-885-5p	1.17 × 10^−6^	1.15 × 10^−8^	6.085013	9.5279	2.680327
hsa-miR-320c	1.17 × 10^−6^	1.24 × 10^−8^	−6.069353	9.4545	−1.62488
hsa-miR-181a	1.67 × 10^−6^	1.97 × 10^−8^	−5.974887	9.014	−1.790673

### 3.2 Overexpression of miR-146b-3p alleviated cerulein-induced cell viability inhibition and apoptosis promotion in AR42J cells

**Figure 2:**
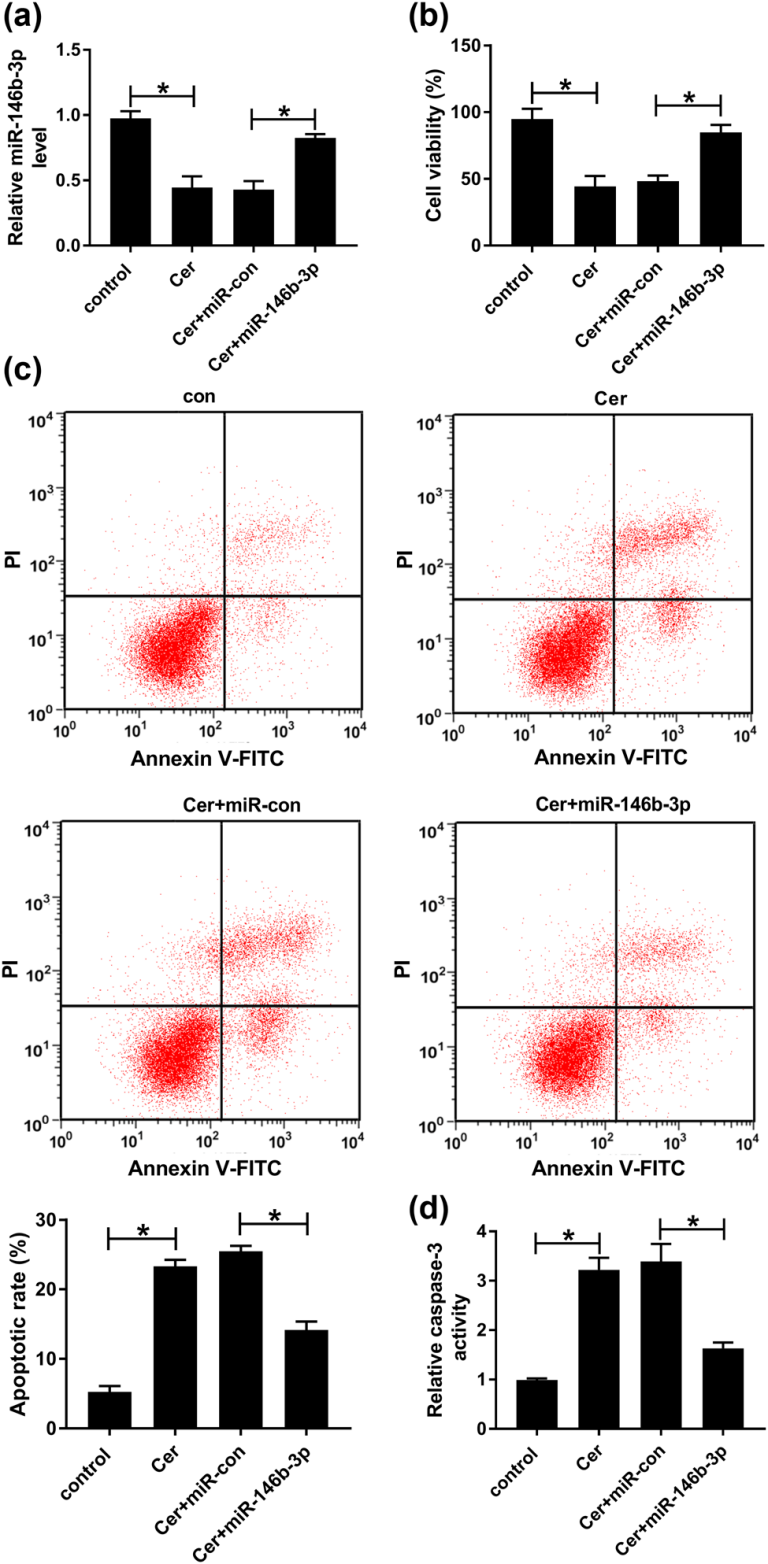
Cerulein-mediated anti-viability and pro-apoptosis effects in AR42J cells were prominently relieved by miR-146b-3p overexpression. AR42J cells were transfected with or without miR-con mimic or miR-146b-3p mimic and then exposed to 10 nmol/L cerulein for 24 h. (a) MiR-146b-3p level was detected by qRT-PCR in treated cells. (b) Cell viability was measured by CCK-8 assay. (c) Cell apoptosis was evaluated by flow cytometry. (d) The caspase-3 activity was determined using a caspase-3 activity assay kit. Blot was representative of *n* = 3. Cer: cerulein. *P < 0.05.

Cerulein, a potent inducer of the AP model, has been widely used to establish the AP cell model [23,24]. The data of qRT-PCR revealed that in contrast to the negative control, cerulein treatment led to a remarkable reduction in the level of miR-146b-3p in AR42J cells (Figure 2a). Then, we determined the impact of cerulein on cell viability and apoptosis. CCK-8 assays showed that compared with the control group, cerulein strikingly weakened cell viability (Figure 2b). The analyses of flow cytometry revealed that cerulein strongly promoted cell apoptosis (Figure 2c). Moreover, cerulein resulted in increased activity of caspase-3 in AR42J cells (Figure 2d).

To understand the role of miR-146b-3p in AP, we manipulated its expression in cerulein-treated AR42J cells. As shown in Figure 2a, the transfection of miR-146b-3p mimic significantly abolished cerulein-mediated miR-146b-3p diminishment (Figure 2a). Further analyses demonstrated that the increased level of miR-146-3p remarkably abrogated cerulein-mediated anti-viability (Figure 2b) and pro-apoptosis (Figure 2c) effects. In addition, miR-146b-3p overexpression highly reversed the enhancement of caspase-3 activity of cerulein in AR42J cells (Figure 2d).

### 3.3 Overexpression of miR-146b-3p ameliorated cerulein-induced inflammation enhancement in AR42J cells

**Figure 3:**
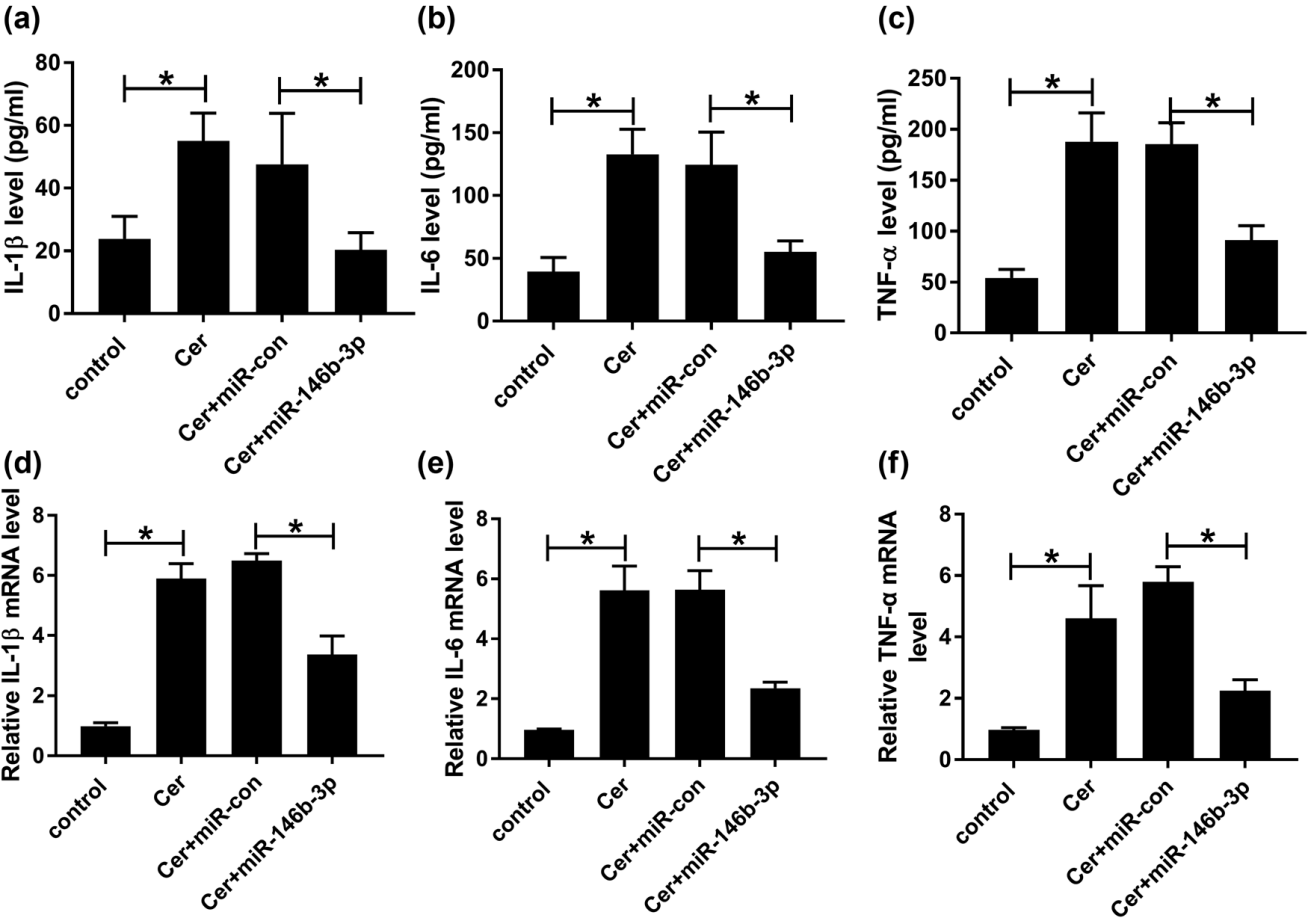
The enhanced influence of cerulein on AR42J cell inflammation was reversed by miR-146b-3p overexpression. AR42J cells were transfected with or without miR-con mimic or miR-146b-3p mimic and then exposed to 10 nmol/L cerulein for 24 h, followed by the determination of IL-1β, IL-6, and TNF-α levels using a corresponding assay kit (a–c), and their mRNA levels by qRT-PCR (d–f). Blot was representative of *n* = 3. Cer: cerulein. *P < 0.05.

Next, we analyzed the effect of miR-146b-3p on the inflammatory response in cerulein-induced AR42J cells. ELISA and qRT-PCR assays revealed that cerulein led to a striking elevation in the levels of pro-inflammatory cytokines (IL-1β, IL-6, and TNF-α) compared with the negative control (Figure 3a–f). Moreover, the increased level of miR-146b-3p significantly abrogated the enhancement of cerulein on IL-1β, IL-6, and TNF-α expressions (Figure 3a–f).

### 3.4 Anxa2 is directly targeted by miR-146b-3p

**Figure 4:**
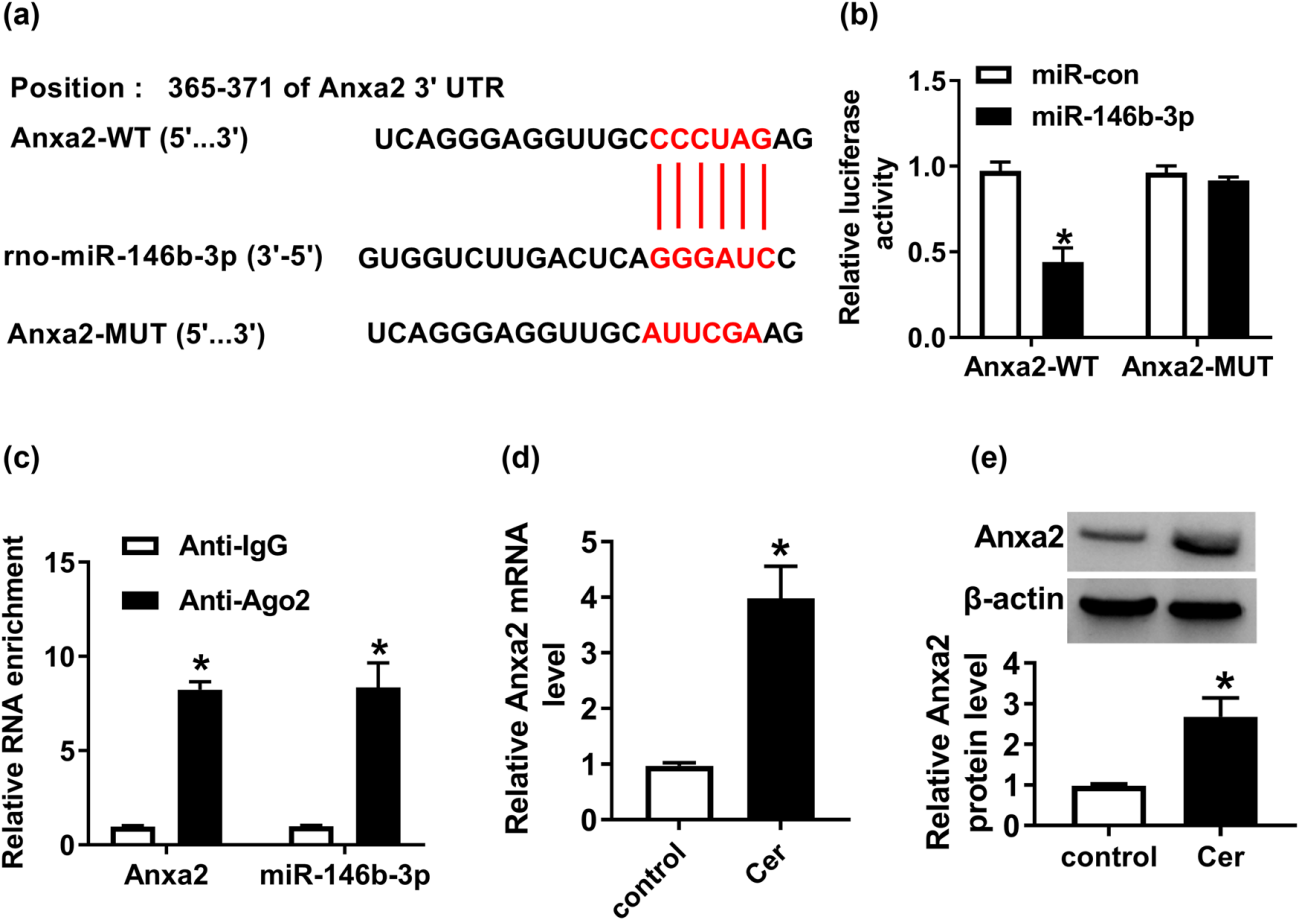
Anxa2 was a direct target of miR-146b-3p in AR42J cells. (a) Schematic of the miR-146b-3p binding sites within Anxa2 3′-UTR and the mutant in the target sequence. (b) Relative luciferase activity was detected in AR42J cells cotransfected with Anxa2 3′-UTR wild-type luciferase reporter (Anxa2-WT) or Anxa2 3′-UTR mutant-type luciferase reporter (Anxa2-MUT) and miR-146b-3p mimic or miR-con mimic. Blot was representative of *n* = 3. (c) Lysates of AR42J cells were incubated with anti-Ago2 or anti-IgG antibody, and then the enrichment of Anxa2 and miR-146b-3p was assessed by qRT-PCR. Blot was representative of *n* = 3. (d and e) Anxa2 mRNA and protein levels were measured in cerulein-treated AR42J cells. Blot was representative of *n* = 3. Cer: cerulein. *P < 0.05.

After demonstrating that miR-146b-3p overexpression alleviated cerulein-induced cell injury, we further explored how miR-146b-3p achieved it. MiRNAs exert biological function by regulating the expression of target mRNAs [25]. Therefore, we performed a detailed analysis of the molecular targets of miR-146b-3p. Using the online software TargetScan v.7.1, a putative complementary sequence for miR-146b-3p was predicted within the 3′-UTR of Anxa2 mRNA (Figure 4a). To determine whether Anxa2 was a direct target of miR-146b-3p, we carried out the dual-luciferase reporter assays using the Anxa2 3′-UTR luciferase reporter. The reporter construct and miR-146b-3p overexpression produced a significant downregulation in luciferase activity (Figure 4b). To verify whether the miR-146b-3p binding sites were required for this effect, a mutant Anxa2 3′-UTR reporter, in which all six predicted complementary sites were mutated, was tested. Notably, the mutant no longer elicited such an effect (Figure 4b). MiRNAs are present in the cytoplasm in the RNA-induced silencing complex, which also contains Ago2 protein [20]. Thus, we conducted RIP experiments using an anti-Ago2 antibody. Compared with the anti-IgG control, the enrichment levels of Anxa2 and miR-146b-3p were synchronously elevated by anti-Ago2 antibody (Figure 4c), implying the endogenous interaction between miR-146b-3p and Anxa2 in AR42J cells. In addition, cerulein caused a significant upregulation of Anxa2 expression at both mRNA and protein levels in AR42J cells (Figure 4d and e).

### 3.5 Anxa2 is a functionally important target of miR-146-3p in regulating cerulein-induced AR42J cell injury

**Figure 5:**
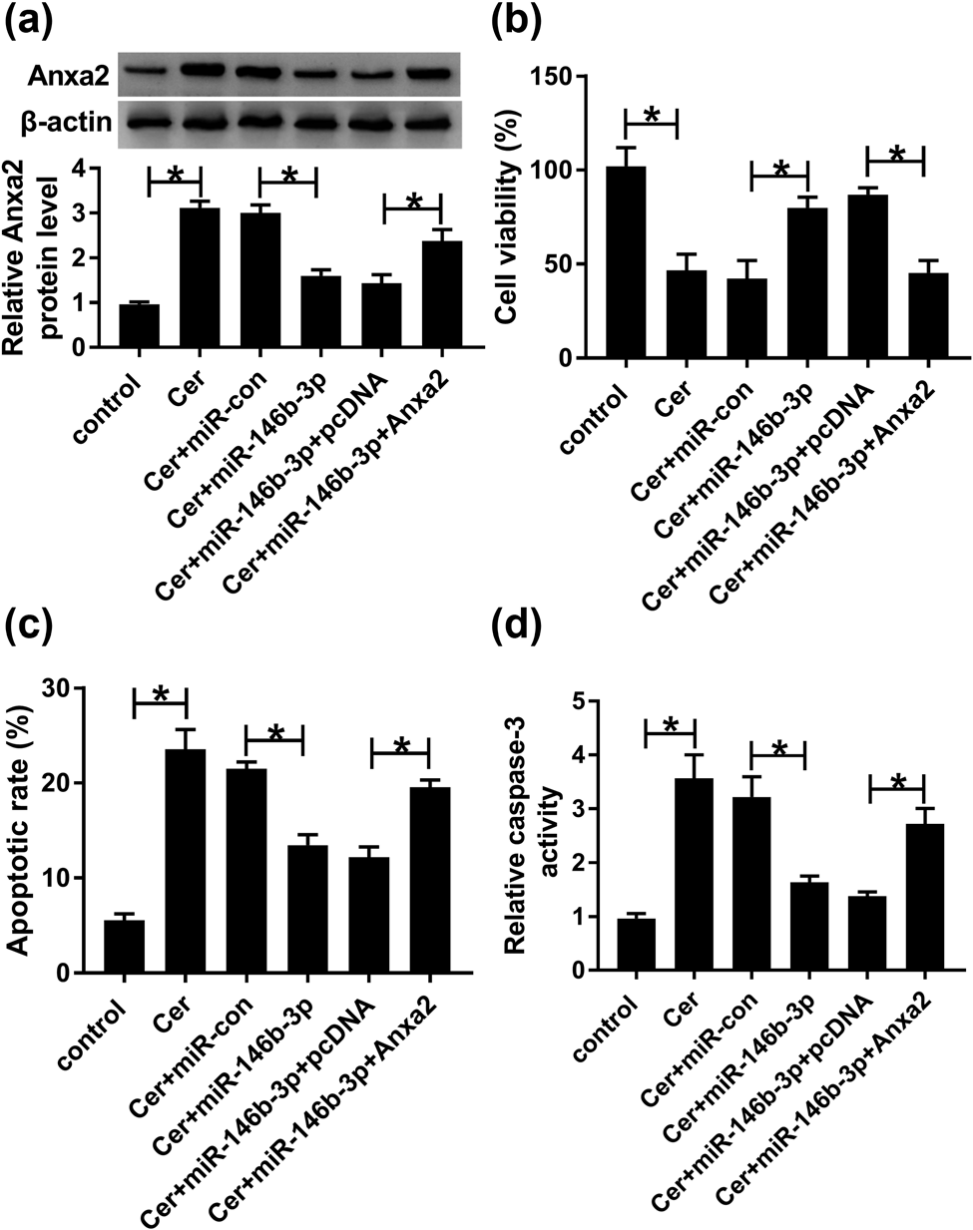
MiR-146b-3p overexpression-mediated pro-viability and anti-apoptosis effects were reversed by the restored Anxa2 level. AR42J cells were transfected with or without miR-con mimic, miR-146b-3p mimic, miR-146b-3p mimic + pcDNA, or miR-146b-3p mimic + Anxa2 and then exposed to 10 nmol/L cerulein for 24 h. (a) Anxa2 protein expression was detected by western blot in treated cells. (b) Cell viability was measured by CCK-8 assay. (c) Cell apoptosis was determined by flow cytometry. (d) Caspase-3 activity was assessed using a caspase-3 assay kit. A representative experiment was shown in triplicate. Cer: cerulein, pcDNA: negative control plasmid, Anxa2: Anxa2 overexpression plasmid. *P < 0.05.

**Figure 6:**
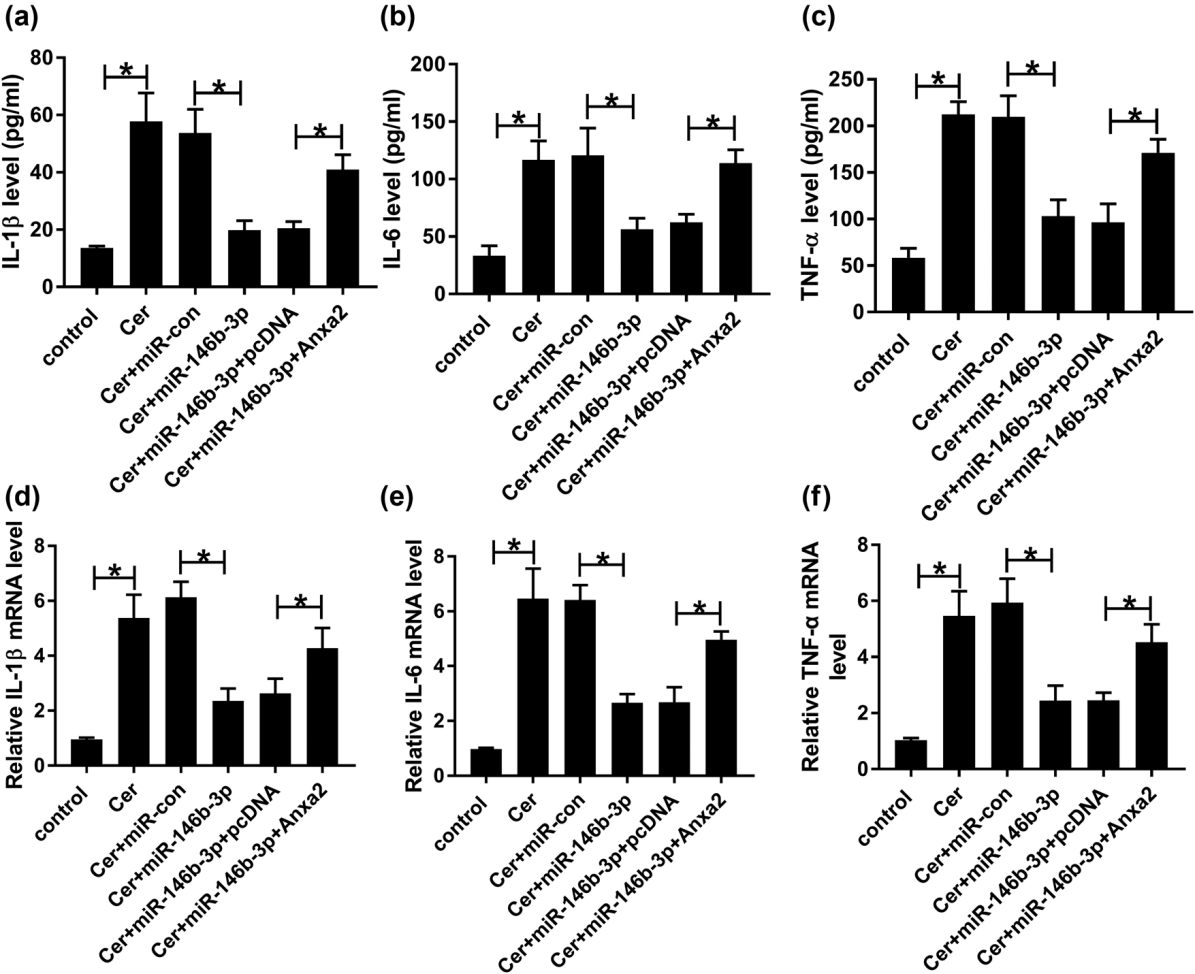
MiR-146b-3p overexpression-mediated anti-inflammation effect was reversed by the restored Anxa2 level. AR42J cells were transfected with or without miR-con mimic, miR-146b-3p mimic, miR-146b-3p mimic + pcDNA, or miR-146b-3p mimic + Anxa2 and then exposed to 10 nmol/L cerulein for 24 h, followed by the detection of L-1β, IL-6, and TNF-α levels using a corresponding assay kit (a–c), and their mRNA levels by qRT-PCR (d–f). Blot was representative of *n* = 3. Cer: cerulein, pcDNA: negative control plasmid, Anxa2: Anxa2 overexpression plasmid. *P < 0.05.

Further, we investigated whether Anxa2 was a functional target of miR-146b-3p in regulating cerulein-induced injury in AR42J cells. As demonstrated by western blot, miR-146b-3p overexpression led to a striking downregulation in the level of Anxa2 protein in cerulein-treated AR42J cells, and this effect was strongly abolished by the co-transfection of Anxa2 overexpression plasmid (Figure 5a). Moreover, compared to the negative control, the restored expression of Anxa2 dramatically abrogated miR-146b-3p overexpression-mediated pro-viability (Figure 5b), anti-apoptosis (Figure 5c and d) effects. Furthermore, Anxa2 expression restoration significantly reversed the repression of miR-146b-3p on IL-1β, IL-6, and TNF-α production (Figure 6a–f).

## 4 Discussion

AP is a sudden inflammatory disorder in the pancreas [26]. MiRNAs have been implicated in the pathogenesis of AP [27,28,29]. The analysis of dysregulated miRNAs in AP using GEO database showed that miR-146b-3p was prominently downregulated in AP samples. Therefore, in this article, we assumed that miR-146b-3p was involved in the pathogenesis of AP and explored its precise actions on AP progression.

Our results first validated the underexpression of miR-146b-3p in the serum samples of AP patients. Interestingly, we first showed that the enforced expression of miR-146b-3p alleviated cerulein-induced injury in AR42J cells, as evidenced by promotion in cell viability and repression in cell apoptosis and inflammation. Caspase-3 activity is closely related to cell apoptosis in the pathological process [30,31]. In the present study, the downregulation of caspase-3 activity also supported the protective role of miR-146b-3p in cerulein-induced cell injury.

Bioinformatic analysis for the molecular targets of miR-146b-3p predicted a putative binding sequence for miR-146b-3p within the 3′-UTR of Anxa2. Anxa2, a Ca^2+^-dependent phospholipid-binding protein, has been identified as a strong oncogenic driver in human cancers [32]. Moreover, Anxa2 plays a crucial role in the tumorigenesis and progression of pancreatic adenocarcinoma [33]. Notably, it was reported that Anxa2 was an important regulator in AP pathogenesis [20]. In this study, we first confirmed that Anxa2 was directly targeted and suppressed by miR-146b-3p in AR42J cells. Our data also demonstrated the significant upregulation of Anxa2 in the serum samples of AP patients and cerulein-treated AR42J cells, consistent with previous work [20]. More importantly, we first showed that Anxa2 was a functionally important target of miR-146b-3p in regulating cerulein-induced AR42J cell injury. Similarly, the examples of the miRNAs protecting against AP progression included miR-148a and miR-193a-5p, which regulated the expression of their targets [9,34]. Conversely, some miRNAs, such as miR-155 and miR-551b-5p, promoted AP progression by silencing target mRNAs [10,35]. Previous reports demonstrated that miR-146-3p associated with the inflammatory response through several inflammation-related signaling pathways, such as p38/MAPK and toll-like receptor 4 pathways [36,37]. Anxa2 was also reported to play an important role in the inflammatory response [38]. A future challenge will be to identify whether the novel mechanism regulates AP progression by modulating inflammation-related pathways. The current work was limited to *in vitro* investigation, and more *in vivo* researches using the AP model will be performed to validate the new mechanism in further work. In addition, the cerulein-induced AP cell model cannot accurately mimic the pathological characteristics of AP, which limited the investigation of the role of miR-146b-3p in AP.

In conclusion, our present study identified that the increased level of miR-146b-3p attenuated cerulein-induced cell injury in AR42J cells at least in part by targeting Anxa2. Our findings highlighted that the miR-146b-3p/Anxa2 axis might be a promising target for AP diagnosis and treatment.

## Appendix

**Table A1:** Primers for PCR

Primers for PCR (5′–3′)		
IL-1β (rat)	Forward	TGATGTTCCCATTAGACAGC
	Reverse	GAGGTGCTGATGTACCAGTT
IL-6 (rat)	Forward	CAAGAGACTTCCAGCCAGTTGC
	Reverse	TTGCCGAGTAGACCTCATAGTGACC
TNF-α (rat)	Forward	TACTGAACTTCGGGGTGATTGGTCC
	Reverse	CAGCCTTGTCCCTTGAAGAGAACC
Anxa2 (human)	Forward	TTGCTGATCGGCTGTATG
	Reverse	GGTAGTCGCCCTTAGTGTC
Anxa2 (rat)	Forward	CACCTCCAGAAAGTGTTCGAAAG
	Reverse	GGCTTGTTCTGAATGCACTGAA
GAPDH (rat)	Forward	TGAAGGTCGGTGTCAACGGATTTGGC
	Reverse	CATGTAGGCCATGAGGTCCACCAC
GAPDH (human)	Forward	GAATGGGCAGCCGTTAGGAA
	Reverse	AAAAGCATCACCCGGAGGAG
